# High-resolution CT findings of pulmonary infections in patients with hematologic malignancy: comparison between patients with or without hematopoietic stem cell transplantation

**DOI:** 10.1007/s11604-022-01260-7

**Published:** 2022-03-14

**Authors:** Yoshie Kunihiro, Nobuyuki Tanaka, Reo Kawano, Toshiaki Yujiri, Kazuhiro Ueda, Toshikazu Gondo, Taiga Kobayashi, Tsuneo Matsumoto, Katsuyoshi Ito

**Affiliations:** 1 Department of Radiology, Yamaguchi University Graduate School of Medicine, 1-1-1 Minamikogushi, Ube, Yamaguchi 755-8505 Japan; 2 Department of Radiology, National Hospital Organization Yamaguchi-Ube Medical Center, 685 Higashikiwa, Ube, Yamaguchi 755-0241 Japan; 3 Center for Integrated Medical Research, Hiroshima University Hospital, Kasumi 1-2-3 Minami-ku, Hiroshima, Hiroshima 734-8551 Japan; 4 Division of Endocrinology, Metabolism, Hematological Science and Therapeutics, Yamaguchi University Graduate School of Medicine, 1-1-1 Minamikogushi, Ube, Yamaguchi 755-8505 Japan; 5 Department of General Thoracic Surgery, Graduate School of Medical and Dental Sciences, Kagoshima University, 8-35-1, Sakuragaoka, Kagoshima, Kagoshima 890-8520 Japan; 6 Division of Pathology, Fujisawa City Hospital, 2-6-1 Fujisawa, Fujisawa, Kanagawa 251-8550 Japan

**Keywords:** X-ray computed tomography, Pneumonia, Hematopoietic stem cell transplantation

## Abstract

**Purpose:**

To evaluate the high-resolution CT (HRCT) findings of pulmonary infections in patients with hematologic malignancy and compare them between patients with or without hematopoietic stem cell transplantation (HSCT).

**Materials and methods:**

A total of 128 patients with hematologic malignancy and pulmonary infection were included in this study. The diagnoses of the patients consisted of bacterial pneumonia (37 non-HSCT cases and 14 HSCT cases), pneumocystis pneumonia (PCP) (29 non-HSCT cases and 11 HSCT cases), and fungal infection other than PCP (20 non-HSCT cases and 17 HSCT cases). Two chest radiologists retrospectively evaluated the HRCT criteria and compared them using chi-squared tests and a multiple logistic regression analysis.

**Results:**

According to the multiple logistic regression analysis, nodules were an indicator in HSCT patients with PCP (*p* = 0.025; odds ratio, 5.8; 95% confidence interval, 1.2–26.6). The centrilobular distribution of nodules was the most frequent (*n* = 4, 36%) in HSCT patients with PCP. A mosaic pattern was an indicator of PCP in both HSCT and non-HSCT patients. There were no significant differences in other infections.

**Conclusion:**

The mosaic pattern could be an indicator of PCP in both HSCT and non-HSCT patients. Nodules with centrilobular distribution might be relatively frequent HRCT findings of PCP in HSCT patients.

## Introduction

Pulmonary infections are the common life-threatening complications in immunocompromised patients with hematologic malignancy. In addition, hematopoietic stem cell transplantation (HSCT) is performed to treat hematologic malignancy and is performed after high-dose chemotherapy or radiation therapy, which is a risk factor for severe opportunistic infections and pulmonary infections after HSCT [1, 2]. An early diagnosis of pulmonary infections is essential to develop an appropriate treatment strategy for these patients.

The differential diagnosis of pulmonary infections in immunocompromised patients could be established with the help of high-resolution CT (HRCT), especially for bacterial pneumonia, pneumocystis pneumonia (PCP), and fungal infection other than PCP [3]. However, some of the characteristic HRCT findings different according to the various immunocompromised statuses. The characteristic HRCT findings of pulmonary infection could be different between HSCT and non-HSCT patients with hematologic malignancy. To our knowledge, no previous studies have evaluated the differences in the HRCT findings of pulmonary infections between HSCT and non-HSCT patients. The purpose of this study was to evaluate and compare the HRCT findings of pulmonary infections (bacterial pneumonia, PCP, and fungal infection other than PCP) in patients with hematological malignancy who were managed with or without HSCT.

## Materials and methods

The institutional review board of our institution approved this study. The requirement for informed consent was waived due to the retrospective design.

### Patients

A total of 128 patients were included in this study from January 1990 to December 2015. There were 78 men and 50 women, and mean age was 49.6 (standard deviation [SD], 17.9; age range 5–77) years.

The diagnoses of the patients consisted of bacterial pneumonia (37 non-HSCT cases and 14 HSCT cases), PCP (29 non-HSCT cases and 11 HSCT cases), and fungal infection other than PCP (20 non-HSCT cases and 17 HSCT cases). Fungal infection other than PCP consisted of pulmonary aspergillosis and pulmonary candidiasis in this study. The exclusion criteria were as follows: (1) the presence of co-infection, (2) immunocompromised state due to a condition other than hematologic malignancy, and (3) other pulmonary abnormalities that could cause difficulty in evaluating HRCT (e.g., interstitial pneumonia, radiation pneumonitis, lung cancer, severe emphysema, bronchial asthma, and graft versus host disease). Diagnoses for infections were established by sputum culture (*n* = 30), bronchoalveolar lavage (BAL) or transbronchial biopsy (TBLB) (*n* = 32), serum marker (*n* = 35 including β-D-glucan > 31 pg/mL for PCP [4], *n* = 16; *Aspergillus* antigen, *n* = 19), blood culture (*n* = 22), autopsy (*n* = 7), urinary antigen test (*n* = 1), and surgical lung biopsy (*n* = 1).

The mean periods between transplantation and CT examination date were 482 (SD, 539; range 18–1652) days in bacterial pneumonia, 347 (SD, 309; 62–1224) days in PCP, and 263 (SD, 330; 13–1338) days in fungal infection other than PCP.

The purpose of this study was to evaluate the HRCT findings of pulmonary infections (bacterial pneumonia, PCP, and fungal infection other than PCP) in patients with hematologic malignancy and to compare them between HSCT and non-HSCT patients. First, we evaluated the difference between each infection in HSCT and non-HSCT patients. Next, we identified the significant indicator for the differentiation of each infection in HSCT or non-HSCT patients. Although the subjects recruited for this study were previously reported in another study that included 345 subjects and which compared HRCT findings among infectious diseases [3], this study was focused on cases with hematologic malignancy and performed with the specific goal of comparing the differences in the HRCT findings of pulmonary infections (bacterial pneumonia, PCP, and fungal infection other than PCP) between HSCT and non-HSCT patients. Therefore, the purpose of the current study differs from that of the previous report [3].

### HRCT examinations

CT examinations at our institution were performed using one of the following CT scanners: TCT-900S (Canon Medical Systems Corporation), Somatom Plus 4, Volume Zoom, Somatom Definition, and Somatom Sensation 64 (Siemens Healthineers Systems). The CT scans were obtained at suspended end-inspiratory effort in the supine position without intravenous contrast material injection. Regarding examination by the TCT-900S, HRCT of the region showing abnormal findings was obtained at 2-mm collimation after obtaining conventional 10-mm collimation scans at contiguous 10 mm intervals. The CT images obtained by the TCT-900S scanner during the early period of data collection were viewed on hard copy films. As for the other multi-slice CT scanners, after contiguous 10-, 7-, or 5-mm section images were reconstructed through the chest, additional HRCT images consisting of 1 or 2 mm collimation were reconstructed at 1-, 2-, 5-, or 10-mm intervals through the abnormal findings. The scanning parameters were 120 or 140 kVp and 160–250 effective mAs for all CT scanners. All image data, with the exception of the TCT-900S data, were interfaced directly to our picture archiving and communication system (PACS) (ShadeQuest, Yokogawa Medical Solutions Corp.). The PACS displayed the image data on monitors (three monitors, 1280 × 1080 matrix, 8-bit viewable gray-scale) for blind reading. All images on the monitors or the hard copy films for the TCT-900 s were used to view both the lung (window width, 1500 or 1750 HU; window level, − 600 or − 700 HU) and mediastinum (window width, 250–400 HU; window level, 40–50 HU).

### Interpretation of the HRCT images

The following HRCT findings were coded as present or absent: (a) consolidation (Con); (b) ground-glass attenuation (GGA); (c) crazy-paving pattern; (d) mosaic pattern; (e) nodules; (f) CT-halo sign; (g) tree-in-bud pattern; (h) bronchial wall thickening; (i) interlobular septal (ILS) thickening; (j) hilar or mediastinal lymph node (LN) enlargement; and (k) pleural effusion. Regarding the HRCT findings of (a), (b), (e) and the overall lesion, the extent of lesions within the entire lung field was graded (0 = 0%, 1 = 1–25%, 2 = 26–50%, 3 = 51–75%, and 4 = 76–100%).

According to GGA or Con, the distribution was classified as segmental, nonsegmental, or lobular. The nodules were classified as micro (< 3 mm), small (3–10 mm), or large (> 10 mm) according to their size and their distribution was also classified as centrilobular, perilymphatic, or random.

As for the overall lesion, the distribution was classified axially as central, peripheral, diffuse, or indeterminate and craniocaudally as upper, lower, diffuse, or indeterminate. Finally, one predominant HRCT pattern was recorded as follows: small nodule, large nodule, diffuse GGA, segmental GGA/Con, non-segmental GGA/Con, or bronchial wall or ILS thickening pattern.

Two chest radiologists (15 and 28 years of experience, respectively) evaluated the HRCT images in random order without any knowledge of the patients’ clinical information except for the fact that all patients had hematologic malignancies. Discordant results between the radiologists were resolved by consensus.

### Statistical analysis

The mean age of the patients was calculated for each infection. The sex and HRCT finding patterns were compared between the HSCT and non-HSCT groups using a chi-squared test as an independent test for each infection. The extent of lesions was finalized as the average of the grades allocated by the two radiologists. The age and the extent of the HRCT findings were compared using a *t* test. Interobserver agreement between the two radiologists was calculated as the kappa value (κ) for the aforementioned HRCT findings from (a) to (k) and as the intraclass correlation coefficient (ICC) for the extent of the lesion, and was rated as follows: slight (0.00–0.20), fair (0.21–0.40), moderate (0.41–0.60), substantial (0.61–0.80), or almost perfect (0.81–1.00). Multiple logistic regression analyses were conducted to identify significant indicators for differentiation between each infection in HSCT and non-HSCT patients. In addition, multiple logistic regression analyses were also conducted to identify significant indicators for the differentiation of each infection from other infections, for example, between bacterial pneumonia and other infections (the combination of PCP and fungal infection other than PCP) in HSCT and non-HSCT cases. The forward selection (likelihood ratio) method was used for the multiple logistic regression analyses, and all variables. including parametric factors, were included. The area under the curve (AUC) of each model was calculated. The statistical analyses were performed using the SPSS software program (version 22.0, IBM). P values of < 0.05 were considered to indicate statistical significance.

## Results

**Fig. 1:**
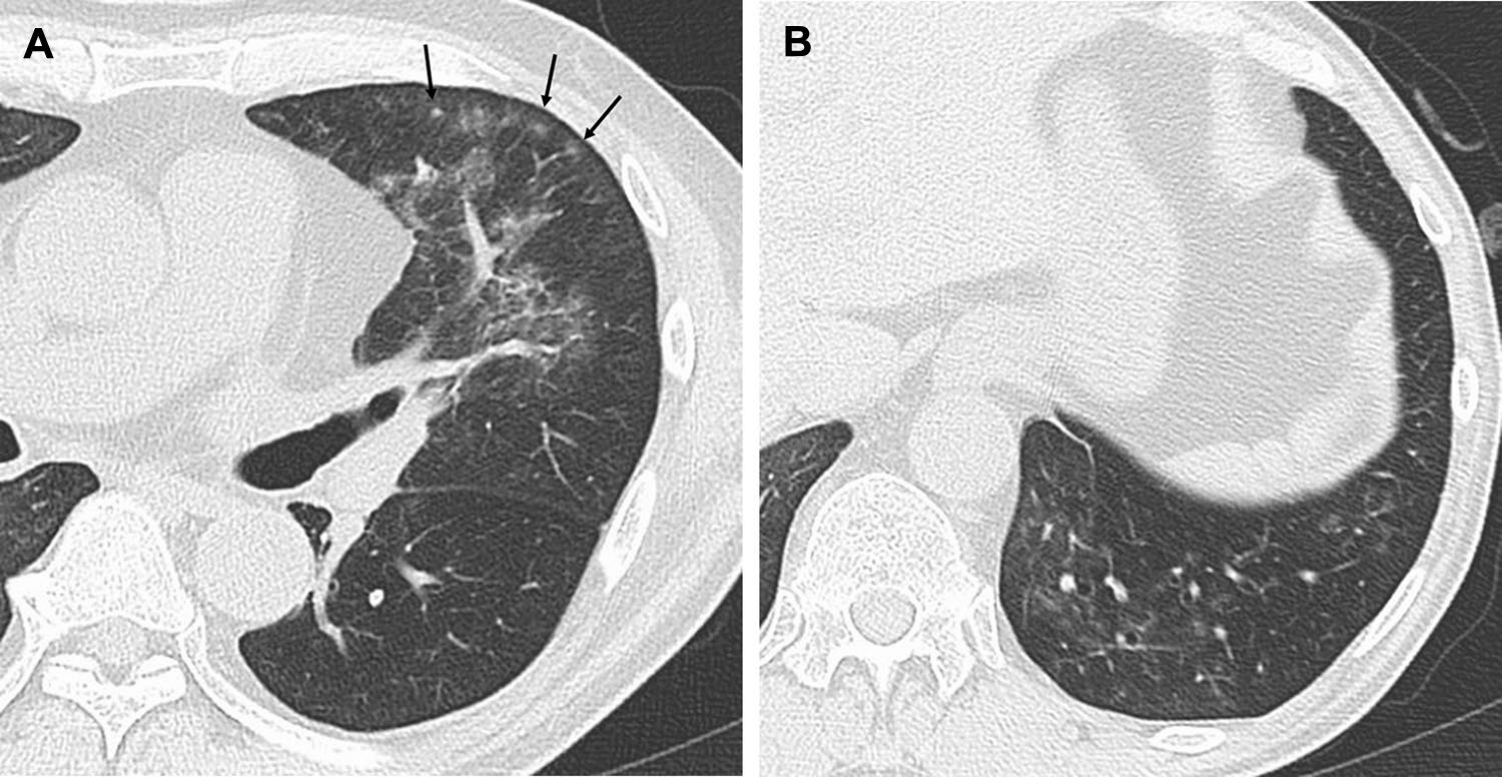
A 59-year-old woman with pneumocystis pneumonia under treatment for malignant lymphoma after hematopoietic stem cell transplantation. **A** High-resolution CT shows ground-glass attenuation with small nodules with centrilobular distribution (arrows). **B** High-resolution CT shows small ground-glass nodules

The HSCT patients were significantly younger than the non-HSCT patients for each of the three infections (*p* < 0.05) (Table 1). The interobserver agreement of the HRCT findings was slight to substantial (0.15–0.72). For PCP, nodules were more frequent in HSCT patients (55%) (Fig. 1) in comparison to non-HSCT patients (17%) (*p* = 0.027). The centrilobular distribution was the most frequent (36%) in HSCT patients with PCP. There were no significant differences in the findings of bacterial pneumonia and fungal infection other than PCP between HSCT patients and non-HSCT patients (Table 1). A multiple logistic regression analysis showed that nodules were an indicator for HSCT patients (*p* = 0.025; odds ratio [OR], 5.8; 95% confidence interval [CI] 1.2–26.6) with PCP.

**Table 1:** Pulmonary infections in patients with and without HSCT

	Bacterial pneumonia		*p* value	PCP		*p* value	Fungal infection other than PCP		*p* value	Interobserver agreement (95% CI)
	non-HSCT (*n* = 37)	HSCT (*n* = 14)		non-HSCT (*n* = 29)	HSCT (*n* = 11)		non-HSCT (*n* = 20)	HSCT (*n* = 17)		
Age (SD)	57.5 (13.8)	34.6 (13.9)	< 0.001^a^	52.2 (16.0)	39.6 (18.8)	0.042^a^	54.4 (20.2)	40.9 (17.0)	0.034^a^	
Man, *n* (%)	25 (68)	8 (57)	0.352^b^	15 (52)	4 (36)	0.385^b^	12 (60)	14 (82)	0.138^b^	
HRCT findings										
Consolidation, *n* (%)	29 (78)	11 (79)	0.653^b^	16 (55)	3 (27)	0.115^b^	11 (55)	8 (47)	0.630^b^	0.48 (0.33, 0.60)
Con-extent (SD)	1.0 (0.6)	0.9 (0.7)	0.507^a^	0.7 (0.7)	0.3 (0.6)	0.129^a^	0.6 (0.6)	0.5 (0.6)	0.723^a^	0.55 (0.32, 0.69)
GGA, *n* (%)	34 (92)	13 (93)	0.700^b^	29 (100)	11 (100)	–	16 (80)	11 (65)	0.251^b^	0.55 (0.42, 0.66)
GGA-extent (SD)	1.4 (0.8)	1.5 (0.8)	0.823^a^	3.2 (0.8)	2.8 (1.1)	0.175^a^	1.2 (0.8)	0.9 (0.8)	0.280^a^	0.72 (0.42, 0.85)
GGA-crazy-paving, *n* (%)	15 (41)	4 (29)	0.430^b^	14 (48)	5 (46)	0.873^b^	4 (20)	4 (24)	0.553^b^	0.55 (0.35, 0.69)
GGA-mosaic, *n* (%)	6 (16)	2 (14)	0.619^b^	23 (79)	6 (55)	0.122^b^	2 (10)	1 (6)	0.562^b^	0.41 (0.18, 0.58)
GGA/Con predominance, *n* (%)						0.275^b^			0.863^b^	0.52 (0.26, 0.56)
Consolidation	20 (54)	5 (36)	0.117^b^	0 (0)	1 (9)		6 (30)	5 (29)		
GGA	11(30)	8 (57)		29 (100)	10 (91)		9 (45)	5 (29)		
Equal	4 (11)	0 (0)		0 (0)	0 (0)		2 (10)	1 (6)		
GGA/Con distribution, *n* (%)			0.679^b^			0.247^b^			0.471^b^	0.30 (0.28, 0.64)
Segmental	26 (70)	8 (57)		1 (3)	0 (0)		10 (50)	8 (47)		
Non-segmental	4 (11)	2 (14)		1 (3)	2 (18)		5 (25)	3 (18)		
Lobular	5 (14)	3 (21)		27 (93)	9 (82)		2 (10)	0 (0)		
Nodules, *n* (%)	21 (57)	9 (64)	0.626^b^	5 (17)	6 (55)	0.027^b)^	19 (95)	14 (82)	0.242^b^	0.35 (0.19, 0.49)
Nodule-extent (SD)	0.7 (0.8)	0.9 (0.8)	0.502^a^	0.3 (0.8)	0.6 (0.6)	0.278^a^	1.4 (0.7)	1.1 (0.7)	0.333^a^	0.54 (0.52, 0.55)
Nodule size, *n* (%)			0.692^b^			0.676^b^			0.968^b^	0.35 (0.29, 0.74)
Micro	8 (22)	2 (14)		1 (3)	2 (18)		3 (15)	2 (12)		
Small	6 (16)	3 (21)		3 (10)	2 (18)		6 (30)	5 (29)		
Large	7 (19)	4 (29)		1 (3)	2 (18)		10 (50)	7 (41)		
Nodule distribution, *n* (%)			0.809^b^			0.652^b^			0.752^b^	0.62 (0.50, 0.85)
Centrilobular	12 (32)	5 (36)		3 (10)	4 (36)		6 (30)	6 (35)		
Perilymphatic	1 (3)	1 (7)		0 (0)	0 (0)		1 (5)	1 (6)		
Random	8 (22)	3 (21)		2 (7)	2 (18)		12 (60)	7 (41)		
Nodules with halo, *n* (%)	7 (19)	5 (36)	0.185^b^	1 (3)	1 (9)	0.479^b^	10 (50)	7 (41)	0.591^b^	0.30 (0.13, 0.45)
Nodules with TIB, *n* (%)	6 (16)	4 (29)	0.268^b^	0 (0)	2 (18)	0.071^b^	3 (15)	4 (24)	0.404^b^	0.45 (0.31, 0.58)
Bronchial wall thickening, *n* (%)	21 (57)	9 (64)	0.626^b^	3 (10)	3 (27)	0.196^b^	8 (40)	8 (47)	0.666^b^	0.69 (0.53, 0.79)
ILS thickening, *n* (%)	14 (38)	5 (36)	0.889^b^	11 (38)	7 (64)	0.135^b)^	1 (5)	2 (12)	0.438^b^	0.16 (− 0.05, 0.36)
Axial distribution, *n* (%)			0.640^b^			0.343^b^			0.069^b^	0.15 (− 0.05, 0.28)
Inner	2 (5)	2 (14)		2 (7)	0 (0)		0 (0)	0 (0)		
Outer	12 (32)	5 (36)		1 (3)	2 (18)		7 (35)	6 (35)		
Diffuse	6 (16)	1 (7)**		22 (76)	7 (64)		5 (25)	0 (0)		
Indeterminate	17 (46)	6 (43)		4 (14)	2 (18)		8 (40)	11 (65)		
Craniocaudal distribution, *n* (%)			0.783^b^			0.248^b^			0.176^b^	0.33 (0.22, 0.52)
Upper	6 (16)	4 (29)*		0 (0)	0 (0)		2 (10)	2 (12)		
Lower	11 (30)	4 (29)		1 (3)	2 (18)		5 (25)	6 (35)		
Diffuse	4 (11)	1 (7)**		24 (83)	7 (64)		5 (25)	0 (0)		
Indeterminate	16 (43)	5 (36)		4 (14)	2 (18)		8 (40)	9 (53)		
Overall extent (SD)	1.9 (0.8)	2.1 (0.8)	0.377^a^	3.5 (0.6)	3.0 (0.9)	0.060^a^	2.0 (0.7)	1.7 (0.8)	0.389^a^	0.56 (0.06, 0.78)
CT pattern, *n* (%)			0.069^b^			0.411^b^			0.185^b^	0.42 (0.20, 0.60)
Small nodule	1 (3)	4 (29)		2 (7)	0 (0)		5 (25)	4 (24)		
Large nodule	6 (16)	2 (14)		0 (0)	1 (9)		8 (40)	6 (35)		
Diffuse GGA	3 (8)	1 (7)		23 (79)	8 (73)		0 (0)	0 (0)		
Segmental	26 (70)	6 (43)		1 (3)	1 (9)		3 (15)	6 (35)		
Non-segmental	1 (3)	1 (7)		3 (10)	1 (9)		4 (20)	4 (20)		
Bronchial/ILS	0 (0)	0 (0)		0 (0)	0 (0)		0 (0)	0 (0)		
LN swelling, *n* (%)	3 (8)	4 (29)	0.358^b^	7 (24)	3 (10)	0.432^b^	3 (15)	3 (18)	0.276^b^	0.29 (0.12, 0.44)
Effusion, *n* (%)	12 (32)	5 (36)	0.889^b^	7 (24)	3 (10)	0.329^b^	2 (10)	8 (47)	0.699^b^	0.57 (0.36, 0.72)

*SD* standard deviation, *HSCT* hematopoietic stem cell transplantation, *PCP* pneumocystis pneumonia, *GGA* ground-glass attenuation, *Con* consolidation, *TIB* tree-in-bud, *ILS* interlobular septum, *LN* lymph node,

*Significantly higher (adjusted standard residuals > 1.96) in groups, **Significantly lower (adjusted standard residuals <  − 1.96) in groups

^a^*t* test

^b^Chi-squared test

**Fig. 2:**
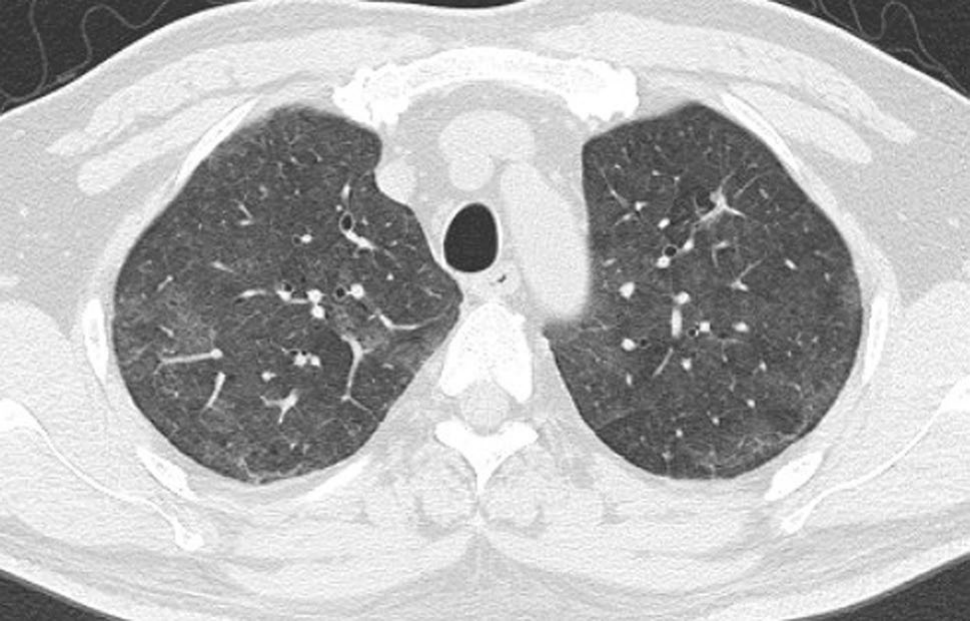
A 52-year-old man with pneumocystis pneumonia during treatment for malignant lymphoma without hematopoietic stem cell transplantation. High-resolution CT shows extensive ground-glass attenuation with a mosaic pattern. Nodules and bronchial wall thickening are not observed

Among non-HSCT patients (Table 2), the following indicators for differentiation among the infectious groups were identified: the absence of a mosaic pattern (*p* = 0.006; OR, 4.5; 95% CI 1.5–13.3) and the presence of bronchial wall thickening (*p* = 0.008; OR, 3.8; 95% CI 1.4–10.1) for bacterial pneumonia; the presence of a mosaic pattern (*p* < 0.001; OR, 15.9; 95% CI 3.9–64.4) and the absence of nodules (*p* = 0.012; OR, 6.0; 95% CI 1.5–24.6) and bronchial wall thickening (*p* = 0.006; OR, 10.2; 95% CI 1.9–53.5) for PCP (Fig. 2); and the presence of nodules (*p* = 0.001; OR, 29.2; 95% CI 3.7–231.8) for fungal infection other than PCP.

**Table 2:** The multiple logistic regression analysis for non-HSCT patients (*n* = 86)

HRCT findings	Bacterial pneumonia (*n* = 37)	Non-bacterial pneumonia (*n* = 49)	Wald value	Odds ratio (95% CI)	*p* value
Mosaic pattern					0.006
Positive	6 (16%)	25 (51%)	7.5		
Negative	31 (84%)	24 (49%)		4.5 (1.5, 13.3)	
Bronchial wall thickening					0.008
Positive	21 (57%)	11 (22%)	7.0	3.8 (1.4, 10.1)	
Negative	16 (43%)	38 (78%)			
	PCP (*n* = 29)	Non-PCP (*n* = 57)	Wald value	Odds ratio (95% CI)	*p* value
Mosaic pattern				15.9 (3.9, 64.4)	< 0.001
Positive	23 (79%)	8 (14%)	15.1		
Negative	6 (21%)	49 (86%)			
Nodules				6.0 [1.5, 24.6]	0.012
Positive	5 (17%)	40 (70%)	6.3		
Negative	24 (83%)	17 (30%)			
Bronchial wall thickening					0.006
Positive	3 (10%)	29 (51%)			
Negative	26 (90%)	28 (49%)	7.5	10.2 (1.9, 53.5)	
	Fungal infection (other than PCP) (*n* = 20)	Non-fungal infection (*n* = 66)	Wald value	Odds ratio (95% CI)	*p* value
Nodules				29.2 (3.7, 231.8)	0.001
Positive	19 (95%)	26 (39%)			
Negative	1 (5%)	40 (61%)	13.3		

*HSCT* hematopoietic stem cell transplantation, *PCP* pneumocystis pneumonia, *CI* confidence interval

The AUC values of the model for bacterial pneumonia, PCP, and fungal infection other than PCP in non-HSCT patients were 0.75, 0.92, and 0.78, respectively (Table 3).

**Table 3:** Sensitivity, specificity, PPV, and NPV of each HRCT finding in non-HSCT patients

	Sensitivity (%)	Specificity (%)	PPV (%)	NPV (%)	AUC
Bacterial pneumonia					0.75
Absence of mosaic pattern	84	51	56	81	
Presence of bronchial wall thickening	57	78	66	70	
PCP					0.92
Presence of mosaic pattern	79	86	74	89	
Absence of nodules	83	70	59	89	
Absence of bronchial wall thickening	90	51	48	88	
Fungal infection other than PCP					0.78
Presence of nodules	95	61	42	98	

*HSCT* hematopoietic stem cell transplantation, *PPV* positive predictive value, *NPV* negative predictive value, *PCP* pneumocystis pneumonia, *AUC* area under the curve

Among HSCT patients (Table 4), the following indicators for differentiation among the infectious groups were identified: the presence of consolidation (*p* = 0.022; OR, 5.7; 95% CI 1.3–25.0) for bacterial pneumonia; the presence of a mosaic pattern (*p* = 0.005; OR, 11.2; 95% CI 2.1–60.1) for PCP; and the absence of GGA (*p* = 0.024; OR, 13.1; 95% CI 1.4–122.2) for fungal infection other than PCP. The mosaic pattern was detected as an indicator for PCP in both HSCT and non-HSCT patients.

**Table 4:** The multiple logistic regression analysis for HSCT patients (*n* = 42)

HRCT findings	Bacterial pneumonia (*n* = 14)	Non-bacterial pneumonia (*n* = 28)	Wald value	Odds ratio (95% CI)	*p* value
Consolidation			5.2	5.7 (1.3, 25.0)	0.022
Positive	11 (79%)	11 (39%)			
Negative	3 (21%)	17 (61%)			
	PCP (*n* = 11)	Non-PCP (*n* = 31)	Wald value	Odds ratio (95% CI)	*p* value
Mosaic pattern			7.9	11.2 (2.1, 60.1)	0.005
Positive	6 (55%)	3 (10%)			
Negative	5 (46%)	28 (90%)			
	Fungal infection (other than PCP) (*n* = 17)	Non-fungal infection (*n* = 25)	Wald value	Odds ratio (95% CI)	*p* value
GGA			5.1	13.1 (1.4, 122.2)	0.024
Positive	11 (65%)	24 (96%)			
Negative	6 (35%)	1 (4%)			

*HSCT* hematopoietic stem cell transplantation, *PCP* pneumocystis pneumonia, *GGA* ground-glass attenuation, *CI* confidence interval

The AUC values of the model for bacterial pneumonia, PCP, and fungal infection other than PCP in HSCT patients were 0.70, 0.72, and 0.66, respectively (Table 5).

**Table 5:** Sensitivity, specificity, PPV, and NPV of each HRCT finding in HSCT patients

	Sensitivity (%)	Specificity (%)	PPV (%)	NPV (%)	AUC
Bacterial pneumonia					0.70
Presence of consolidation	79	61	50	85	
PCP					0.72
Presence of mosaic pattern	55	90	67	85	
Fungal infection other than PCP					0.66
Absence of GGA	35	96	86	69	

*HSCT* hematopoietic stem cell transplantation, *PPV* positive predictive value, *NPV* negative predictive value, *PCP* pneumocystis pneumonia, *GGA* ground-glass attenuation, *AUC* area under the curve

## Discussion

Our study showed that there are both similarities and differences in HRCT findings between HSCT patients and non-HSCT patients.

In PCP, nodules were observed significantly more frequently in HSCT patients than in non-HSCT patients. There were no significant differences in the HRCT findings of HSCT and non-HSCT patients in bacterial pneumonia and fungal infection other than PCP. On the other hand, the model for each infection between HSCT and non-HSCT patients was identified in this study. The AUC values of the model for PCP were the highest among these infections (0.92 in non-HSCT and 0.72 in HSCT).

In PCP, the mosaic pattern was an indicator in both HSCT and non-HSCT patients in our study. In addition, the absence of nodules was an indicator in non-HSCT patients. Previous studies reported that the mosaic pattern was frequently observed in PCP, while nodules were an unusual finding in PCP [3, 5–7]. Nodules have shown to represent granulomas and could be observed in human immunodeficiency virus (HIV) infection, and hematopoietic and solid malignancies [8]. The granulomatous reaction is related to host factors, including immune reconstitution disease and the recovery of partial immune efficiency following the interruption of corticosteroid treatment [9]. HSCT patients with PCP could show nodules because of their relatively strong immunosuppression and immune reconstitution state under treatment.

For bacterial pneumonia, the bronchial wall thickening was an indicator in HSCT patients and the consolidation was an indicator in non-HSCT patients. Bronchial wall thickening and consolidation are common characteristics of bacterial pneumonia [3, 10–12].

For fungal infection other than PCP, the nodules could be an indicator in non-HSCT patients. This result supports the findings of a previous study [3]. Invasive aspergillosis shows nodules with the halo sign in the early phase, and cavitary lesions with the air crescent sign in the late phase [13–15]. Nodules and cavitation are also common findings in cryptococcosis [16] though patients with cryptococcosis were not included in this study. Small nodules with centrilobular or random distribution are common in candidiasis [17, 18]. The absence of GGA could be an indicator in HSCT patients; however, GGA is a nonspecific finding and its sensitivity was low in the present study (35%).

The present study was associated with some limitations. First, this study was retrospective in nature; thus, the CT protocols and diagnostic procedures were diverse. However, the minimum CT protocols required to obtain the presented result were satisfied. Second, the reliability of the diagnosis may be controversial in patients without pathological confirmation. However, patients with infections due to other organisms or co-infections were excluded from our study, and diagnoses were strongly supported by serum markers or urinary antigen tests. Third, the interobserver agreement score was low of some criteria. The kappa values of mosaic pattern and nodules were 0.41 and 0.35, respectively. These results could affect the reliability of this study. These findings and ILS thickening could be overestimated by one radiologist. However, the discordant results between radiologists were resolved by consensus. The other criteria were not simple binary variables; thus, therefore, inter-individual variations may be noted. Forth, the patients with other infections such as cytomegalovirus pneumonia were not included in this study, because the number was limited. The purpose of this study was to focus on cases with hematologic malignancy based on the previous study, which identified the significant indicators especially for bacterial pneumonia, PCP, and fungal infection other than PCP among immunocompromised patients [3].

In conclusion, HRCT findings should be evaluated with consideration of the patient’s state irrespective of whether they underwent HSCT. In PCP, while the mosaic pattern could be the indicator of PCP in both HSCT and non-HSCT patients, nodules might be relatively frequent in HSCT patients than in non-HSCT patients.
